# Potential biodegradation of polycyclic aromatic hydrocarbons (PAHs) and petroleum hydrocarbons by indigenous fungi recovered from crude oil-contaminated soil in Iran

**DOI:** 10.1038/s41598-023-49630-z

**Published:** 2023-12-13

**Authors:** Maryam Fallahi, Mohammadsaeed Sarempour, Amir Mirzadi Gohari

**Affiliations:** 1 Department of Plant Pathology, Faculty of Agricultural Sciences and Engineering, College of Agriculture and Natural Resources, University of Tehran, Daneshkadeh Ave., Karaj, Iran; 2 Environmental Science and Engineering, Islamic Azad University, Tehran North Branch, Tehran, Iran

**Keywords:** Environmental impact, Microbiology

## Abstract

A total of 265 fungal individuals were isolated from soils exposed to heavy oil spills in the Yadavaran oil field in Iran to discover indigenous fungal species with a high potential to biodegrade petroleum hydrocarbon pollutants. Morphological and molecular identification of obtained fungal species led to their assignment into 16 genera and 25 species. *Alternaria* spp. (78%), *Fusarium* spp. (5%), and *Cladosporium* spp. (4%) were the most common genera, along with *Penicillium* spp., *Neocamarosporium* spp., *Epicoccum* sp., *Kotlabaea* sp., *Aspergillus* sp., *Mortierella* sp., and *Pleurotus* sp. A preliminary screening using the DCPIP indicator revealed that approximately 35% of isolates from *Alternaria*, *Epicoccum*, *Neocamarosporium*, *Cladosporium*, *Fusarium*, *Stachybotrys*, *Penicillium*, and *Stemphylium* demonstrated promising tolerance to crude oil. The best-performing isolates (12 fungal individuals) were further investigated for their capacity to mineralize a mixture of four polycyclic aromatic hydrocarbons (PAH) for 47 days, quantified by GC–MS. Eventually, two top-performing isolates, namely 5c-12 (*Alternaria tenuissima*) and 3b-1 (*Epicoccum nigrum*), were applied to petroleum-contaminated soil. The GC–MS analysis showed that 60 days after inoculation, these isolates successfully degraded more than 70% of the long-chain hydrocarbons in the soil, including C8-C16 n-alkanes, C36 n-alkane, and Pristane. This study introduces two fungal species (5c-12 and 3b-1) with high potential for biodegrading petroleum compounds and PAHs, offering promising prospects for the decontamination of oil-contaminated soil.

## Introduction

Petroleum, often referred to as crude oil, is a naturally occurring mixture of hydrocarbon and non-hydrocarbon molecules, including paraffinic, naphthenic, and aromatic compounds, found deep beneath the Earth's surface. Soil contaminated by petroleum is a critical and challenging environmental issue in various locations, especially in soil where contamination tends to accumulate significantly^1^. This contamination includes a diverse group of lipophilic organic compounds known as polycyclic aromatic hydrocarbons (PAHs), composed of two or more fused aromatic rings^2^. PAHs occur naturally in crude oil and gasoline and are often found in environmental samples, with more than 100 different PAHs identified, some of which have been shown to be mutagenic and carcinogenic to humans, animals, and plants^3,4^. PAHs can have a destructive impact on soil functions and disrupt the soil's microbiome due to their persistence and resistance to degradation under natural conditions^5–7^. Low-molecular-weight (LMW) PAHs, containing two or three aromatic rings, such as naphthalene, anthracene, fluorene, acenaphthene, and phenanthrene, are known for their acute toxicity. In contrast, high-molecular-weight (HMW) PAHs, which contain four or more rings, including chrysene, pyrene, benzo(a)pyrene, and fluoranthene, are largely considered genotoxic^8^. PAHs have the ability to covalently bind to DNA, RNA, and proteins, and the extent of this covalent interaction with DNA determines their carcinogenicity^9^.

The extensive extraction, transportation, and consumption of crude oil enhanced the chance of oil spills unintentionally into the environment, harming terrestrial and aquatic ecosystems and posing a serious threat to human and animal health^10,11^. For example, the international energy agency [IEA] (2022) announced that global oil demand was rising by 5.5 million b/d and 3.3 million b/d in 2021 and 2022, respectively. Moreover, a much higher global demand for crude oil is anticipated in 2050. This rise in demand has led to a significant increase in environmental pollution caused by PAHs, particularly due to the growing consumption of biodiesel. Consequently, there is an urgent need for efficient and environmentally-safe approaches to mitigate the negative and severe impacts of oil spills on the environment.

Various remediation technologies, including physical, chemical, and biological approaches, are developed to rehabilitate the polluted sites^12,13^. Physico-chemical methods for removing toxic chemicals, while effective, can be expensive and may lead to new contamination by transferring pollutants from one phase to another. However, despite their effectiveness, these techniques are often impractical for large-scale use^14,15^.

In recent decades, bioremediation, a process that involves using biological agents with high tolerance to the toxicity of hydrocarbons, has garnered significant interest as an environmentally friendly and cost-effective method for cleaning up contaminated sites^16–18^. Mycoremediation, a form of bioremediation that leverages the metabolic capabilities of fungi to decontaminate polluted environments, is a natural biological process that results in the reduction, detoxification, degradation, mineralization, or conversion of more harmful pollutants into less toxic forms using fungal agents^19,20^. Fungi are a vital part of soil microbiota that plays a central role in nutrient cycling and biotic interaction with other microorganisms, such as bacteria, by expanding their mycelial networks. Fungal genomes harbor essential genes encoding diverse metabolic pathways and secreting a variety of extracellular enzymes, conferring them the capacity to grow and develop on a broad range of natural and synthetic substances and degrade diverse hydrocarbons or complex molecules to small ones^21^. The use of indigenous fungi in mycoremediation provides a unique opportunity to reduce environmental pollutants due to their robust morphology and diverse metabolic capabilities. Fungi produce various extracellular and intracellular enzymes, including catalases, laccases, peroxidases, and cytochrome P450 monooxygenases, which are involved in detoxification and the biodegradation of toxic organic compounds. Additionally, fungi can penetrate contaminated soil through their hyphae structure, reaching toxic and recalcitrant compounds and using them as sources of energy and carbon for growth and reproduction^22,23^.

Previous studies indicated that indigenous fungi belonging to the genera *Cladosporium*, *Alternaria*, *Fusarium*, *Trichoderma*, *Aspergillus*, *Penicillium*, *Paecilomyces*, *Coriolus*, *Pycnoporus*, *Pleurotus*, *Cephalosporium*, *Mucor*, *Fomitopsis*, and *Daedalea* are responsible for degrading of PAHs and diesel oil in the soil and aquatic environments^19,24,25^. Currently, scientists have made significant attempts to identify fungi adapted to oil-contaminated environments that can degrade PAHs, the recalcitrant fractions of petroleum and crude oil, since they are critical components in impacting the success rate of bioremediation^26^. Presently, most native fungal isolates were investigated to determine their ability to degrade individual PAHs, which existed in crude oil, but comprehensive information on the biodegradation level of total petroleum hydrocarbons (TPHs) is scarce.

This study had the objective of isolating and characterizing fungal isolates collected from both oil-contaminated and non-contaminated sites. Our initial screening process involved 256 isolated individuals and employed a colorimetric method to assess their capability to biodegrade petroleum. Subsequently, we conducted morphological assessments to classify isolates into genera, followed by a colorimetric assay to identify those with PAH degradation potential. Eventually, the 65 isolates selected for molecular sequencing represent a subset chosen based on their PAH degradation capabilities within prevalent genera identified through the initial morphological classification. This analysis revealed two particularly promising fungi, namely, 5c-12 (*Alternaria tenuissima*) and 3b-1 (*Epicoccum nigrum*), demonstrating a significant degradation of PAHs. Following this discovery, we further evaluated these two top-performing isolates by introducing them to sterilized soil that had been artificially contaminated with petroleum. The subsequent GC/MS analysis confirmed that these isolates were capable of degrading over 70% of the long-chain PAHs present in the soil assay.

## Materials and methods

### Fungal isolation

Soils contaminated with crude oil from the Yadavaran oil field in the western region of Khuzestan province, located in the south of Iran, were collected to identify specific fungi with a high capability for degrading crude oil. Sampling was conducted on a sunny day with a temperature of 32 °C and a humidity level of 65%. Samples weighing 500 g were taken from one site with non-contaminated soil (site 5) and four sites within the contaminated area (sites 1 to 4). Sampling was performed randomly at three depths (A: 0 cm, B: 20 cm, and C: 40 cm) at each site. The collected subsamples were thoroughly mixed to create one sample from each site/depth. These samples were sieved through a 2.5 mm mesh to remove stones, and the sieved soils were air-dried at room temperature for use in the fungal isolation procedure. Fungal isolation was carried out using a dilution plate technique. One gram of soil sample was added to a 0.05% Water Agar (WA) medium, prepared by mixing 0.5 g of agar in 1 L of water and autoclaving it at 121 °C for 15 min. Subsequently, 1 ml of a 10-times diluted soil suspension was spread onto Potato Dextrose Agar (PDA), Malt Extract Agar (MEA), and Water Agar (WA) media. These plates were then incubated at 25 °C for 7 days, and the developed colonies on each plate were transferred to fresh plates. The experimental research adhered to relevant institutional, national, and international guidelines and legislation.

### Fungal identification

Fungal identification involved a combination of morphological characterization, encompassing features such as conidiomata, conidiophores, conidiogenous cells, and culture traits, complemented by DNA sequencing of marker genes. We employed various taxonomic keys to morphologically distinguish each fungal isolate^27–29^. For molecular identification, genomic DNA was extracted from fresh mycelia of each fungal individual growing on PDA using the CTAB (cetyltrimethylammonium bromide detergent) method, as previously described^30^. To check the quality and quantity of DNA extracts, a gel electrophoresis technique, and a Nanodrop ND-2000 Spectrophotometer were utilized (Thermo Scientific, Wilmington, DE, USA). The internal transcribed spacer 1 (ITS1), ITS2 regions and the 5.8 S ribosomal DNA (rDNA) were amplified by using universal primers ITS1 (5′-TCCGTAGGTGAACCTGCGG-3′) and ITS4 (TCCTCCGCTTATTGATATGC-3′)^31^. The PCR mixture, totaling 50 μl, consisted of 2 μl of DNA (10 ng), 5 μl of 10 × MgCl2, 1 μl of each deoxynucleoside triphosphate (5 mM), 0.25 μl of Taq polymerase (ROCHE Diagnostics GmbH, No. 14647428) at a concentration of 5 U, and 2.5 μM for each primer (5 pmol). The PCR cycling conditions were as follows: an initial denaturation for 5 min at 95 °C, followed by 30 standard cycles. Each cycle involved denaturation at 94 °C for 30 s, primer annealing at 58 °C for 50 s, and primer extension at 72 °C for 1 min. The final extension step was performed at 72 °C for 10 min. Subsequently, the PCR products were electrophoresed in a 0.8% agarose gel in Tris–borate-EDTA buffer at pH 8 for 1 h at 80 V and visualized using a gel imager (Gel Doc EZ Imager; Bio-Rad Laboratories, Austria). All PCR products underwent a cleaning process with ExoSap-It (USB Corporation, Cleveland, OH) following the manufacturer's instructions. Sequencing was performed using a BigDye Terminator (version 3.1) cycle sequencing kit and a capillary sequencer (3500 genetic analyzers; Applied Biosystems, Life Technologies Corporation, Carlsbad, CA). The obtained nucleotide sequences were edited using Pro Chromas version 1.7.6^32^ and Editseq version 5.01^33^. To assess their similarity to previously reported sequences stored in the GenBank database (http://www.ncbi.nlm.nih.gov/blast/), the Basic Local Alignment Search Tool (BLAST)^34^ was employed.

### Screening the biodegradability of petroleum hydrocarbons (PHs) in vitro

A colorimetric assay using the 2,6-dichlorophenolindophenol (DCPIP) indicator was employed to assess the capacity of the selected fungal isolates in biodegrading petroleum hydrocarbons (PHs). DCPIP is an enzyme-catalyzed redox electron acceptor that starts as dark blue in its oxidized form and turns colorless when reduced during the biodegradation process. This change occurs due to a molecular structural shift from a double to a single carbon–nitrogen bond^35^. To achieve this objective, the Bushnell-Haas broth (BH) medium was utilized, consisting of MgSO4 (0.2 g/l), CaCl2 (0.02 g/l), KH2PO4 (1 g/l), K2HPO4 (1 g/l), FeCl3 (0.05 g/l), and NH4NO3 (1 g/l). Additionally, Tween 80 (0.1%), DCPIP (2%), and petroleum (1%) were incorporated into the BH medium. The reduction in color was measured to determine if the fungi could utilize petroleum as their sole carbon source for growth. Each culture medium, supplemented with DCPIP, was incubated at 26 °C in a shaker incubator set at 180 rpm for 47 days. This assay was conducted with three biological samples and independently repeated twice. Negative controls consisted of cultures with DCPIP and petroleum but without fungal cultures.

### GC/MS analysis

To quantify both the components of PAHs (Phenanthrene, Acenaphthene, Benzo(a)pyrene, Pyrene) and the total petroleum hydrocarbon (TPH) in BH medium and soil samples, respectively, gas chromatography-mass spectrometry (GC–MS) analysis was performed. For the analysis of PAHs in the BH medium, 250 ml Erlenmeyer flasks were filled with 50 ml of BH medium containing a mixture of PAHs (0.2 g/L, with 0.05 g/L of each PAH), DCPIP (2%), and 0.5 cm^2^ of fresh mycelium. Negative controls included BH medium with DCPIP and PAHs but without fungal culture. The extraction of PAHs was carried out using methanol (99.9%) and hexane (99.9%) as solvents. This assay was independently repeated three times per sample. All cultures were incubated for 47 days at 26 °C in a shaker incubator set at 180 rpm. The GC–MS analysis was conducted using an Agilent 7890 N gas chromatograph with an HP-5MS column. The inlet temperature was set at 290 °C. The column's temperature was initially maintained at 60 °C for 1 min, then gradually increased at a rate of 5 °C per minute for 2 min until it reached 290 °C. The injector temperature was held isothermally at 290 °C with a splitless mode for 3 min, and the solvent delay time was set at 5 min. Helium was used as the carrier gas at a flow rate of 1 ml/min. Metabolite identification was based on retention time and fragmentation in comparison to standards.

The percentage degradation of PAHs was calculated using the following equation:

$$ {\text{PAHs degradation }}\left( \% \right) \, = \frac{{CPAHs_{c} {-}CPAHs_{t} }}{{CPAH_{c} }} \times \% 100 $$

where *CPAHs*_*c*_ = Concentration of PAHs in control, *CPAHs*_*t*_ = Concentration of PAHs in each treatment.

### Soil assay

Top-performing candidates (5c-12 and 3b-1), selected based on the results of GC/MS analysis, were subjected to an investigation of their ability to biodegrade PHs in sterilized soil artificially contaminated with petroleum over a 60-day period under laboratory conditions. Before artificial contamination, the physicochemical characteristics and nutritional content of the soil were determined (Table 1), and the soil was autoclaved at 120 °C for 30 min to sterilize it. For optimal degradation, NH4NO3 was added as a nitrogen source, and KH2PO4 was added as a phosphorus source to set the soil C/N/P ratio at 100:10:1. The sterilized soil was artificially contaminated with 10% (v/w) petroleum and 10% (v/w) fungal inoculum. Additionally, 250 mg/L of glucose, serving as a carbon source, was added to each sample every 10 days throughout the assay. After 60 days, the remaining total petroleum hydrocarbon content in the contaminated soil was analyzed using the GC–MS technique and compared to the control soil (contaminated with 10% (v/w) petroleum but without fungal inoculum). For the fungal inoculum, 30 g of wheat grains were placed in a 250 ml Erlenmeyer flask. Subsequently, 13.5 mL of distilled water was added to each flask, and the mixture was left overnight before being autoclaved at 120 °C for 30 min. The flasks containing autoclaved wheat were then inoculated with 5 mm diameter discs of fungal cultures (grown on PDA at 25 °C for 10 days) and incubated at 25 °C for 21 days to promote fungal development.

**Table 1:** Physicochemical characteristics of the studied soil.

Number	Physical and chemical characteristics of the applied soil	
1	PH	7.7
2	EC	9.93
3	Nitrogen (%)	0.65
4	Phosphorus (mg/kg)	531
5	Potassium(mg/kg)	1596
6	Texture	Sandy loam
7	Clay (%)	10
8	Silt (%)	25
9	Sand (%)	65
10	Organic matter (%)	7.44
11	Color	Dark brown

### Statistical analysis

The data analysis was performed using SAS software (version 9.14). To identify significant differences (*p* ≤ 0.01) among the various treatments, a one-way analysis of variance (ANOVA) was conducted. Subsequently, the Duncan multiple comparisons test was employed to compare the means.

## Results

### Fungal identification and Frequency distribution

**Figure 1:**
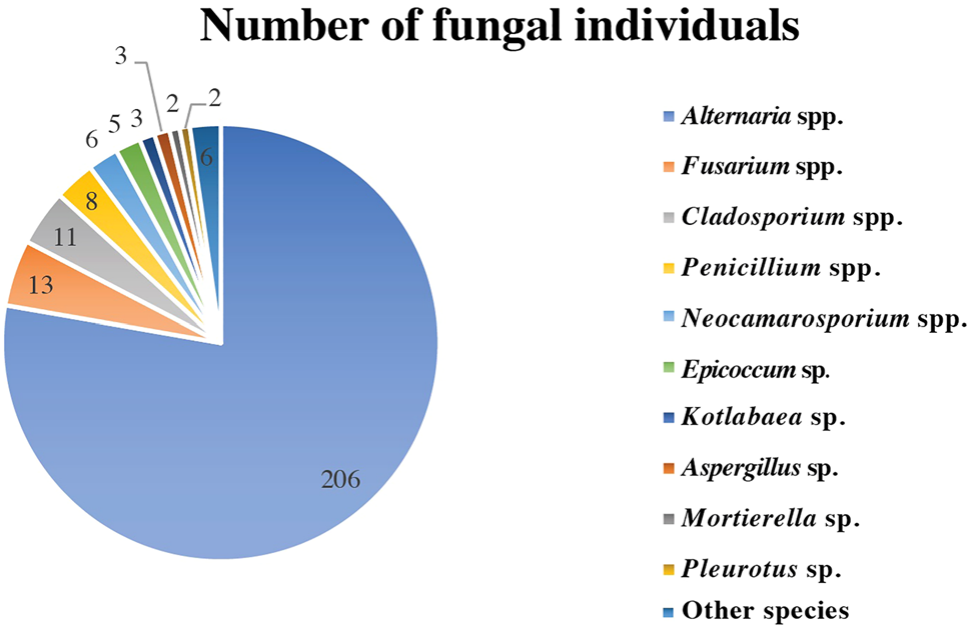
Frequency distribution of various fungal genera isolated from soil samples collected from the Yadavaran oil field in the western region of Khuzestan, Iran, categorized into oil-contaminated (sites 1 to 4) and non-contaminated (site 5) soils. The number of isolates for each genus is presented.

Overall, a total of 265 individual fungal isolates were obtained from the five sampling sites, with 207 isolates collected from crude oil-contaminated sites (sites 1 to 4) and 58 isolates from non-contaminated soil (site 5). These isolates were identified using both morphological and molecular methods, leading to their classification into 16 genera and 25 species (Table 2). To facilitate molecular identification, we initially conducted morphological identification and aimed to choose isolates from different genera. Additionally, among the isolates belonging to prevalent genera, we prioritized those with positive results in colorimetric screening for further molecular identification. This dual selection approach ensured a comprehensive representation of fungal diversity within our study. In total, 65 representative fungal isolates were selected, encompassing a broad spectrum of genera and species. These isolates were then subjected to sequencing for the *ITS* gene, and the resulting sequences were meticulously curated and submitted to GenBank for reference (Table 2). Significantly, the most prevalent genera in this study were *Alternaria* spp. (78%), followed by *Fusarium* spp. (5%), and *Cladosporium* spp. (4%). Additionally, *Penicillium* spp., *Neocamarosporium* spp., *Epicoccum* sp., *Kotlabaea* sp., *Aspergillus* sp., *Mortierella* sp., *Pleurotus* sp., *Botryotrichum* sp., *Galactomyces* sp., *Microascales* sp., *Stachybotrys* sp., *Stemphylium* sp., and *Stolonocarpus* sp. were also identified, though less frequently (Fig. 1). For more detailed information on the number of fungal individuals retrieved from different sampling depths (A, B, and C) and the predominance of specific genera (*Alternaria* spp., *Fusarium* spp., and *Cladosporium* spp.), refer to Table 3.

**Table 2:** Comprehensive data for 65 representative fungal species gathered from soil samples in the Yadavaran oil field, situated in the western region of Khuzestan, Iran.

Isolate code	Fungal species	Location	GenBank accession numbers (ITS)
1b-6	*Alternaria alternata*	Site 1-South of Iran	OP886752
4a-1	*Alternaria alternata*	Site 4-South of Iran	OP881519
1c-4	*Alternaria alternata*	Site 1-South of Iran	OP886762
2c-6	*Alternaria atra*	Site 2-South of Iran	OP881518
2c-4	*Alternaria atra*	Site 2-South of Iran	OP881520
2c-5	*Alternaria atra*	Site 2-South of Iran	OP881521
2c-7	*Alternaria atra*	Site 2-South of Iran	OP886760
4b-8	*Alternaria atra*	Site 4-South of Iran	OP886761
2a-10	*Alternaria chlamydospora*	Site 2-South of Iran	OP881509
3a-4	*Alternaria chlamydospora*	Site 3-South of Iran	OP881514
1a-7	*Alternaria chlamydospora*	Site 1-South of Iran	OP886755
4a-28	*Alternaria chlamydospora*	Site 4-South of Iran	OP881515
2a-11	*Alternaria chlamydospora*	Site 2-South of Iran	OP886757
5a-19	*Alternaria chlamydospora*	Site 5-South of Iran	OP881516
1b-12	*Alternaria chlamydospora*	Site 1-South of Iran	OP886756
5a-29	*Alternaria cumini*	Site 5-South of Iran	OP881522
3a-2	*Alternaria longipes*	Site 3-South of Iran	OP886754
5c-6	*Alternaria molesta*	Site 5-South of Iran	OP881512
4a-16	*Alternaria sorghi*	Site 4-South of Iran	OP886759
5c-12	*Alternaria tenuissima*	Site 5-South of Iran	OP881513
3a-6-1	*Aspergillus terreus*	Site 3-South of Iran	OP886764
3a-3	*Botryotrichum piluliferum*	Site 3-South of Iran	OP881538
2a-5	*Cladosporium* sp.	Site 2-South of Iran	OP886767
3b-4-1	*Cladosporium* sp.	Site 3-South of Iran	OP881517
5a-14	*Cladosporium* sp.	Site 5-South of Iran	OP886765
1a-20	*Cladosporium* sp.	Site 1-South of Iran	OP886766
1c-5	*Cladosporium* sp*.*	Site 1-South of Iran	OP886768
3a-9	*Cladosporium* sp*.*	Site 3-South of Iran	OP881526
2b-5	*Cladosporium* sp.	Site 2-South of Iran	OP881525
3a-47	*Cladosporium* sp.	Site 3-South of Iran	OP881527
1b-3	*Cladosporium* sp*.*	Site 1-South of Iran	OP881524
3b-7	*Cladosporium* sp*.*	Site 3-South of Iran	OP881528
2a-33	*Epicoccum nigrum*	Site 2-South of Iran	OP881529
3b-1	*Epicoccum nigrum*	Site 3-South of Iran	OP886769
4a-12	*Fusarium equiseti*	Site 4-South of Iran	OP886771
4a-7	*Fusarium equiseti*	Site 4-South of Iran	OP886772
4a-15	*Fusarium incarnatum*	Site 4-South of Iran	OP886777
4a-26	*Fusarium equiseti*	Site 4-South of Iran	OP886773
5c-10	*Fusarium incarnatum*	Site 5-South of Iran	OP886774
4a-5	*Fusarium incarnatum*	Site 4-South of Iran	OP886775
2c-2	*Fusarium incarnatum*	Site 2-South of Iran	OP886776
5a-27	*Fusarium solani*	Site 5-South of Iran	OP886770
2a-35	*Galactomyces candidum*	Site 2-South of Iran	OP881530
1a-15	*Kotlabaea* sp.	Site 1-South of Iran	OP881510
4a-13–2	*Kotlabaea* sp.	Site 4-South of Iran	OP886763
4c-2	*Corollospora* sp.	Site 4-South of Iran	OP886780
5c-13	*Mortierella alpina*	Site 5-South of Iran	OP881535
1c-8	*Mortierella alpina*	Site 1-South of Iran	OP886781
1a-42	*Neocamarosporium betae*	Site 1-South of Iran	OP881539
5b-15	*Neocamarosporium phragmitis*	Site 5-South of Iran	OP886778
1c-3	*Neocamarosporium chichastianum*	Site 1-South of Iran	OP881523
1a-6	*Neocamarosporium chichastianum*	Site 1-South of Iran	OP886779
1a-5-2	*Neocamarosporium chichastianum*	Site 1-South of Iran	OP886758
4b-1	*Neocamarosporium obiones*	Site 4-South of Iran	OP881511
3a-46	*Penicillium chrysogenum*	Site 3-South of Iran	OP881531
2a-22	*Penicillium oxalicum*	Site 2-South of Iran	OP881532
4a-35	*Penicillium oxalicum*	Site 4-South of Iran	OP881533
3a-37	*Penicillium oxalicum*	Site 3-South of Iran	OP881534
3a-28	*Pleurotus* sp.	Site 3-South of Iran	OP881536
1a-5-1	*Stachybotrys chartarum*	Site 1-South of Iran	OP886782
3a-20	*Stemphylium* sp.	Site 3-South of Iran	OP886753
3b-8	*Stolonocarpus gigasporus*	Site 3-South of Iran	OP881537

These samples were collected from both oil-contaminated (sites 1 to 4) and non-contaminated (site 5) soils. GenBank accession numbers (ITS) are also provided. The sampling took place in January 2019.

**Table 3:** Enumeration of fungal individuals isolated from soils originating in the Yadavaran oil field, located in the western region of Khuzestan, Iran.

Site of sampling	Number of fungal isolates	Fungal isolates occurrence in each level of sampling (%)			Fungal genus occurrence in each site (%)			
		A	B	C	*Alternaria* spp.	*Fusarium* spp.	*Cladosporium* spp.	Other genus
1	70	80	10	10	81	1	4	14
2	35	80	3	17	83	3	6	8
3	67	86	11	3	79	0	7	13
4	35	88	9	3	51	29	0	20
5	58	64	15	21	84	4	2	10
Total	265	79	10	11	78	5	4	13

The data is categorized based on the contamination status of the soil, which includes oil-contaminated sites (sites 1 to 4) and a non-contaminated site (site 5). The number of fungal individuals is further detailed according to the three sampling depths (A: 0 cm, B: 20 cm, C: 40 cm), and the predominant genera, namely *Alternaria* spp., *Fusarium* spp., and *Cladosporium* spp., are highlighted.

### Biodegradation of petroleum hydrocarbons by the isolated fungi

**Figure 2:**
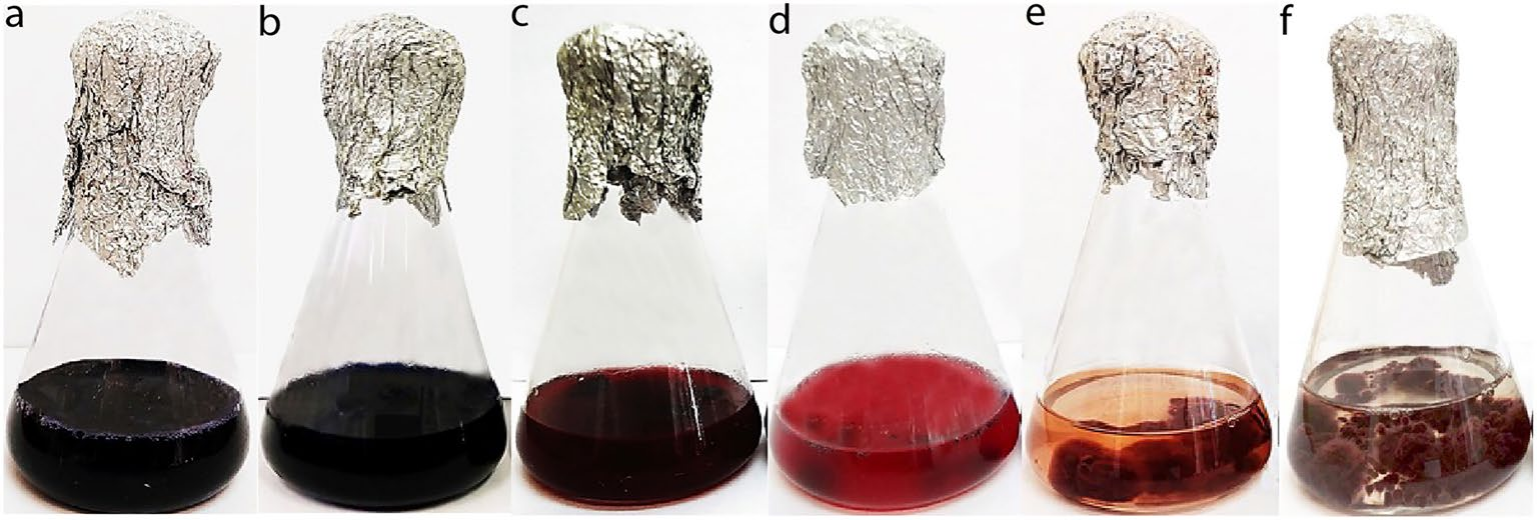
Describing the color and the mass of fungal growth in a BH medium, treated with redox indicator. (**a**) Control sample with dark blue color. (**b**) Isolate 3b-8 (*Stolonocarpus gigasporus*) showed no significant color change and no discernible difference in fungal mass formation compared to the control. (**c**) Isolate 3a-6 (*Aspergillus terreus*) exhibited a minor color change and relatively low fungal mass generation compared to the control (Dark fire brick). (**d**) Isolate 5a-27 (*Fusarium solani*) displayed a moderate color change and moderate fungal mass formation compared to the control (Fire brick). (**e**) Isolate 3b-1 (*Epicoccum nigrum*) demonstrated a significant color change (light fire brick) and substantial fungal mass formation compared to the control. (**f**) Isolate 5c-12 (*Alternaria tenuissima*) exhibited a very pronounced color change (Plain pink) and substantial fungal mass formation compared to the control.

The initial screening process revealed that fungal isolates could be categorized into five groups (Groups I–V) based on their ability to biodegrade petroleum hydrocarbons. This categorization was determined by assessing changes in the color of fungal cultures and the amount of fungal mass produced in the BH medium treated with a redox indicator (Table 4; Fig. 2). Group I included fungal individuals that had no discernible effect on the color of the BH medium, which remained dark purple or blue, and no fungal mass developed. This group consisted of 11% of the investigated isolates, including *Stolonocarpus* sp., *Botryotrichum* sp., *Galactomyces* sp., *Pleurotus* sp., *Mortrierella* sp., and *Microascales* sp. (Table 4). Group II comprised isolates that caused minimal color changes (pinkish purple/dark fire brick) compared to the control and showed limited fungal mass formation. Results indicated that 18% of the examined isolates, including *Aspergillus* sp., *Cladosporium* spp., *Kotlabaea* sp., *Neocamarosporium* sp., and *Alternaria* spp., fell into this category. Group III consisted of isolates that induced moderate color changes (dull pink/maroon/brownish) in the BH medium compared to the control, accompanied by an intermediate fungal mass formation. Our data revealed that 36% of examined isolates belonged to this group, including the majority of *Epicoccum* sp., *Penicillium* sp., *Cladosporium* spp., and *Fusarium* spp. Group IV included isolates that led to a significant color change (light pink or cream/light fire brick) compared to the control and produced substantial fungal mass. Approximately 20% of the studied isolates were categorized into this group, primarily composed of *Alternaria* spp., *Epicoccum* sp., *Neocamarosporium* sp., *Cladosporium* spp., *Fusarium* spp., *Stachybotrys* sp., *Penicillium* spp., and *Stemphylium* sp. Notably, this group featured isolates with the potential for petroleum degradation. Group V encompassed isolates that resulted in distinct color changes (clear pink or colorless) compared to the control and exhibited massive fungal mass formation. Our data confirmed that 15% of the explored isolates, including *Alternaria* spp., *Epicoccum* sp., *Neocamarosporium* sp., and *Fusarium* sp., with pronounced color changes, were placed in this group. The members of this group were identified as promising petroleum-degrading fungi. This screening process indicated that approximately 35% of the isolates (comprising members of both Group IV and Group V) (Table 4) exhibited a significant positive impact on the degradation of petroleum compounds. Therefore, these isolates were considered potential candidates for a quantitative assessment of their capacity to degrade petroleum hydrocarbons. The results of our analysis revealed that isolates obtained from both contaminated and non-contaminated soil are indeed distributed in different groups. For the subsequent assay, we chose 12 individuals for GC–MS analysis, aiming for a comprehensive representation of fungal isolates across diverse performance groups (Group I, II, III, IV, and V). Notably, our selection criteria went beyond the recognized high PHs and PAHs degraders in groups IV and V, encompassing isolates from groups I, II, and III. This inclusive approach sought to thoroughly explore potential variations in degradation capabilities across the entire spectrum of isolates, complementing the qualitative nature of the colorimetric screening. Detailed data, including the distribution of selected individuals from each group, can be found in Supplementary Table 1.

**Table 4:** Grouping of different fungal genera into five categories (Group I to Group V) based on the colorimetric method.

Group I	Group II	Group III	Group IV	Group V
No color change compared to control (dark purple or blue) (11%)	Low color change compared to control (pinkish purple/dark fire brick) (18%)	Moderate color change compared to control (dull pink/fire brick/Brownish (36%)	High color change compared to control (light pink or cream/light fire brick (20%)	Very high color change compared to control (clear pink or colorless (15%)
*Alternaria* (6.5% C)	*Alternaria* (14%C + 5% NC)	*Alternaria* (27%C + 10% NC)	*Alternaria* (12.5%C + 5%NC)	*Alternaria* (15%C + 5%NC)
*Neocamarosporium* (20% NC)	*Neocamarosporium* (20%NC)	*Penicillium* (60%C)	*Neocamarosporium* (20% C)	*Neocamarosporium* (40%C)
*Stolonocarpus* (100%C)	*Cladosporium* (35%NC)	*Cladosporium* (55%C)	*Cladosporium* (10%C)	*Fusarium* (10%C)
*Kotlabaea* (70%C)	*Kotlabaea* (30%C)	*Fusarium*(50%C)	*Fusarium* (30%C + 10%NC)	*Epicoccum* (20% C)
*Botryotrichum* (100%C)	*Aspergillus* (100%C)	*Epicoccum* (60%NC)	*Epicoccum* (20%C)	
*Galactomyces* (100%C)			*Stachybotrys* (100%C)	
*Pleurotus* (100%C)			*Stemphylium* (100%C)	
*Mortrierella* (50%C + 50%NC)			*Penicillium* (40%C)	
*Microascales* (100%C)				

The reported data includes the total percentage of isolates in each group and the percentage of isolates from each genus in each group, obtained from two different sampling sites (*C* contaminated, *NC* non-contaminated). In each flask containing a BH medium and a fungal culture, Tween 80 (0.1%), DCPIP (2%), and petroleum (1%) were added, and the inoculated flasks were kept for 47 days at 26 °C in a shaker incubator set at 180 rpm. Photographs were taken after 47 days post-inoculation and visual observations were documented.

### GC–MS analysis

**Figure 3:**
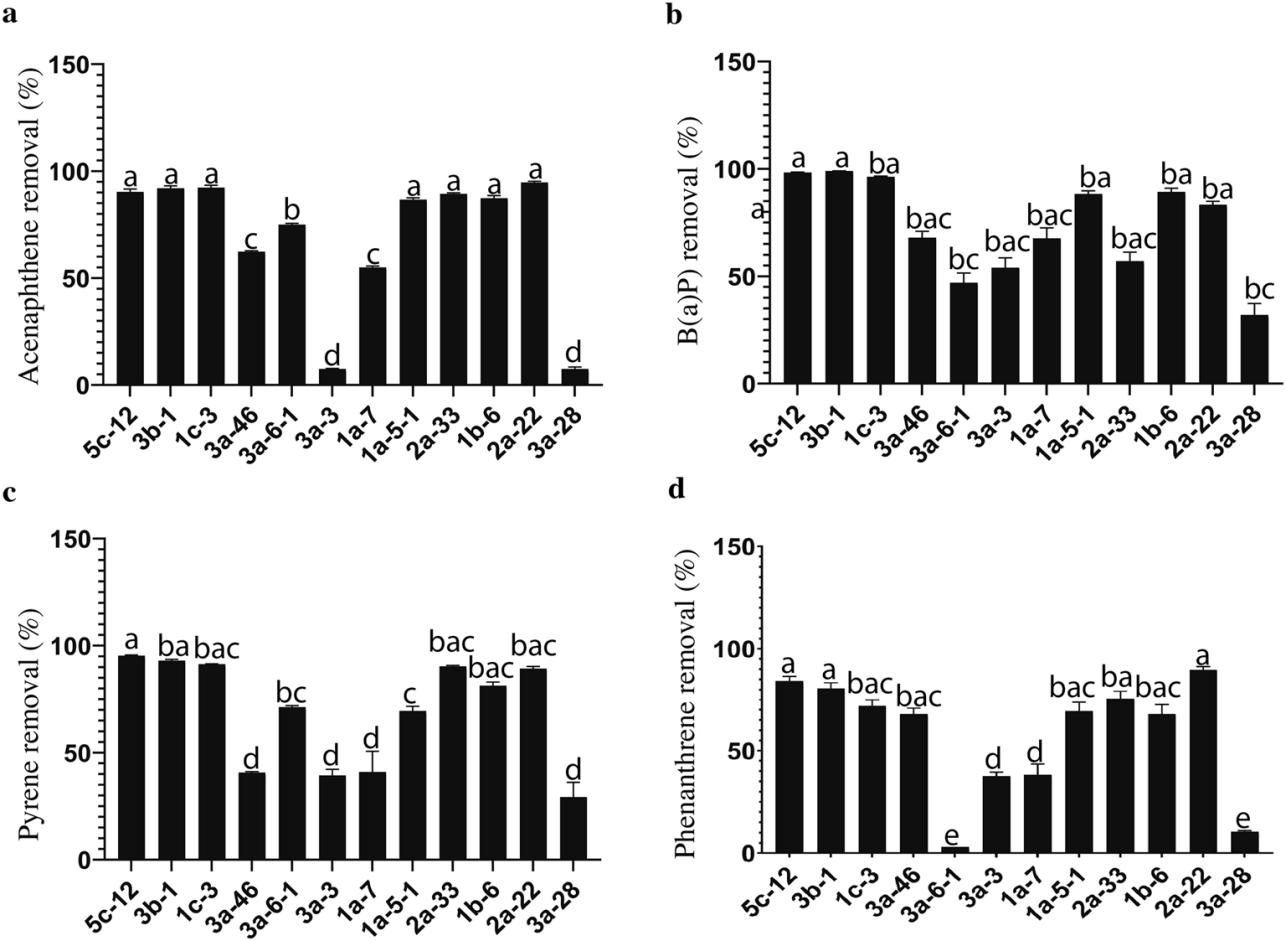
Percentage of Acenaphthene (**a**), Benzo(a)pyrene (B(a)P) (**b**), Pyrene (**c**), and Phenanthrene (**d**) Degraded by Twelve Fungal Isolates: 5c-12 (*Alternaria tenuissima*), 3b-1 (*Epicoccum nigrum*), 1c-3 (Neocamarosporium chichastianum), 3a-46 (*Penicillium chrysogenum*), 3a-6-1 (*Aspergillus terreus*), 3a-3 (*Botrytrichum piluliferum*), 1a-7 (*Alternaria chlamydospora*), 1a-5-1 (*Stachybotrys chartarum*), 2a-33 (*Epicoccum nigrum*), 1b-6 (*Alternaria alternate*), 2a-22 (*Penicillium oxalicum*), and 3a-28 (*Pleurotus* sp.) after 47 days of incubation in a mineral medium inoculated with mycelial plugs of each fungal isolate. The percentage of PAHs degraded by each isolate represents the mean of three replicates, and error bars indicate the standard deviations of means (n = 3). Different lowercase letters denote significant differences (*p* ≤ 0.01) between isolates within a single PAH.

The effectiveness of the fungal isolates in biodegrading petroleum hydrocarbons (PHs) and specific polycyclic aromatic hydrocarbons (PAHs) such as Acenaphthene, Benzo(a)pyrene (B(a)P), Pyrene, and Phenanthrene, as determined by the colorimetric method, was rigorously confirmed through gas chromatography-mass spectrometry (GC–MS) analysis. Notably, not only the fungal isolates from groups IV and V, known for their high PHs and PAHs biodegradation capabilities, but also those from groups I, II, and III, were selected for GC–MS analysis. This precise and sensitive assay revealed that fungal isolates displaying a considerable PAH degradation rate (> 60%) belonged to the genera *Alternaria*, *Epicoccum*, *Neocamarosporium*, *Penicillium*, and *Stachybotrys*. Intriguingly, it was observed that isolates 5c-12 (Attributed to *Alternaria tenuissima*) and 3b-1 (Attributed to *Epicoccum nigrum*) exhibited the capability to degrade the majority of the tested PAHs in a BH medium, and this proficiency was consistent across all the investigated PAHs (Fig. 3). As a result, both isolates were recognized as highly effective remediators and were consequently chosen for further evaluation in a soil assay. Finally, isolate 3a-28 (Attributed to *Pleurotus* sp.) displayed the lowest degradation rate in consuming the four tested PAHs under laboratory conditions.

### In situ assay

**Figure 4:**
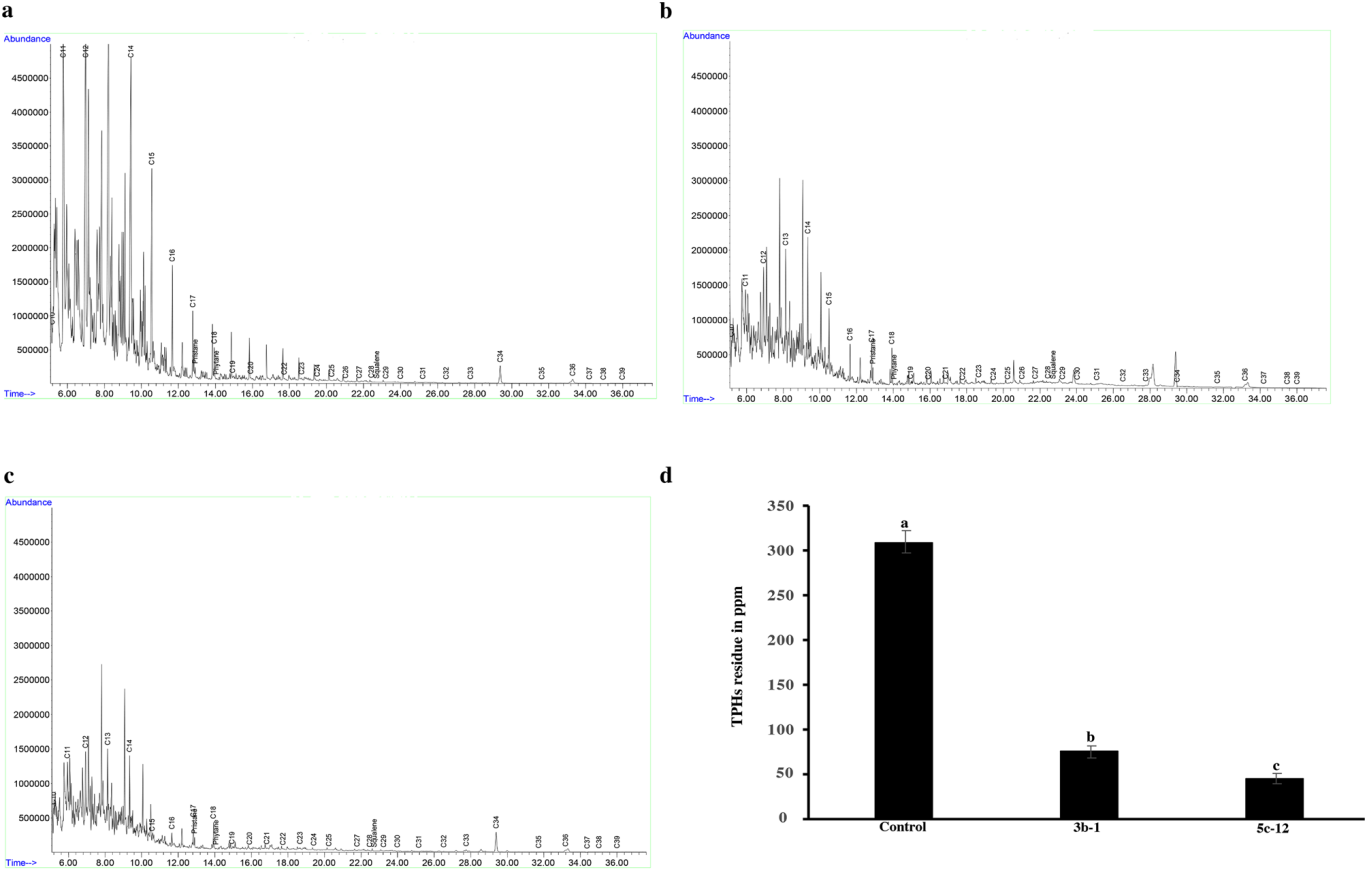
Residual contents of petroleum n-alkanes (C8–C39) after 60 days of incubation in petroleum-contaminated soil, inoculated with fungal strains 5c-12 (*Alternaria tenuissima*), 3b-1 (*Epicoccum nigrum*), and control (without fungi). (**a**) GC–MS chromatograms of total contents of n-alkanes in petroleum-contaminated soil without fungi (**b**), with isolate 3b-1, and isolate 5c-12 (**c**). (**d**) Total petroleum hydrocarbons (TPHs) detected by GC–MS analysis.

Two fungal isolates (5c-12 and 3b-1) were introduced into the soil, following the previously described protocol. This was done to gain a deeper understanding of the capabilities of the highest-performing isolates, which were validated through GC–MS analysis. The aim was to assess their potential to biodegrade petroleum hydrocarbons under *in-situ* conditions using the GC–MS technique. The two isolates under scrutiny exhibited biodegradation activities in soils contaminated with petroleum, as evidenced by the GC–MS fingerprints (Fig. 4a–c). Our soil GC–MS analysis identified 35 compounds, the majority of which fell into the category of aliphatic hydrocarbons. Notably, 10 of these compounds (Supplementary Table 2) showed a significant reduction in the treated soil, which was artificially contaminated by petroleum and subsequently inoculated with targeted isolates. This intervention made a substantial contribution to a 70% reduction in the total petroleum hydrocarbon (TPH) (Fig. 4d). In comparison to the control soil, which remained free from fungal isolates, the soil treated with isolates 5c-12 and 3b-1 witnessed the removal of most petroleum fractions, including C8-C16 and C36 n-alkanes, as well as Pristane (Figure supplementary 1). This attests to the efficacy of the selected isolates in diminishing the levels of specific hydrocarbon compounds and the overall TPH, providing further evidence of their potential in bioremediation efforts.

## Discussion

The release of petroleum and its derivatives poses a severe threat to the natural environment, leading to soil degradation. Recalcitrant organic pollutants, particularly polycyclic aromatic hydrocarbons (PAHs), play a central role in this process^36^. Petroleum products and PAHs, widely used in various industries, have the potential to pollute the environment, water bodies, and seas, with harmful effects on both wildlife and human health due to their carcinogenic properties^2,37^. Mycoremediation has emerged as a promising approach to biodegrade various pollutants in natural ecosystems^38^. Tremendous efforts have been made to isolate organisms, such as fungal individuals degrading highly recalcitrant pollutants, such as PAHs^19,20^ and are currently considered as potential remediators to decontaminate the oil-contaminated soil.

Here, 265 fungal individuals were recovered from soils contaminated by crude oil in the Yadavaran oil field based in the western region of Khuzestan, Iran, where oil extraction, transportation, and oil spill are frequently occurring. Our analysis aimed to identify the isolated fungi, revealing that assigned groups belonging to *Dothideomycetes* (*Alternaria* spp., *Cladosporium* spp., *Neocamarosporium* spp., *Epicoccum* sp., and *Stamphylium* sp.) were frequent inhabitants in the examined soil compared to other groups such as *Sordariomycetes* (*Botryotrichum* sp., *Microascales* sp., *Stachybotrys* sp., and *Stolonocarpus* sp.), *Eurotiomycetes* (*Penicillium* spp. and *Aspergillus* spp.), *Pezizomycetes* (*Kotlabaea* sp.), *Saccharomycetes* (*Galactomyces sp.*), *Mortierellomycetes* (*Mortierella* sp.) and *Agaricomycetes* (*Pleurotus* sp.). This distribution aligns with a previous study that sought to identify culturable hydrocarbonoclastic fungus from South Trinidad's Marac-Moruga mud volcano, indicating the highest combined prevalence (89%) within the *Dothideomycetes* and *Eurotiomycetes* taxonomic groups^39^. Moreover, we observed that the surface layer (A) contained the highest number of fungi, while the number of isolates decreased in the subsurface levels (B and C). These findings align with previous studies reporting a higher fungal frequency in the surface soil layer compared to subsurface levels, likely due to differences in physical, chemical, and microbiological properties across soil horizons^40,41^. It is well documented that different soil horizons have diverse physical, chemical, and microbiological properties. Hence, it can be concluded that an existence of a larger supply of nitrogen and the climatic conditions in this layer may be the central elements causing the increased fungal abundance in the surface layer^42^.

Our in vitro analysis revealed that most of the isolated fungal isolates with significant potential to biodegrade petroleum and PAHs, particularly those with three and four aromatic rings, belonged to the *Dothideomycetes* and *Eurotiomycetes* groups. These isolates were more efficient than other groups in utilizing PAHs and n-alkanes as their sole carbon source (Figs. 2, 3). Notably, the use of DCPIP proved effective in screening the top-performing fungal isolates for petroleum or crude oil biodegradation, a method supported by previous studies^43,44^. However, our findings contrast with a study conducted by Marchand et al.^19^, where fungal isolates from the *Sordariomycetes* group displayed a high potential for PAH degradation in petroleum-contaminated soil in Quebec, Canada, outperforming the *Dothideomycetes* and Mucoromycotina groups.

The fungal individuals suggested for potential application as agents for decontaminating oily soil belonged to the three main fungal phyla, Ascomycota, Basidiomycota, and Mucoromycota. Al-Dhabaan^45^ reported three *Aspergillus* spp. in the collection of 22 fungal isolates recovered from crude oil-contaminated soil of Dhahran in Saudi Arabia with an acceptable degradation efficiency ranging from 47 to 51%. Another study proved that *Trichoderma tomentosum* and *Fusarium oxysporum* isolated from petroleum-contaminated soil could degrade remarkedly all four tested PAHs (anthracene, phenanthrene, fluorene, and pyrene) in the mixture compared to the control^19^. *Aspergillus oryzae* recovered from the shores of the Red Sea, Saudi Arabia, in a study aimed to characterize fungal isolates responsible for degrading crude oil displayed an extraordinary capacity resulting in removing 99% of the crude oil after culturing this isolate in Bushnell–Haas medium for 2 weeks^46^. Benguenab and Chibani^26^ introduced two filamentous fungi (*A. ustus* and *Purpureocillium lilacinum*), isolated from used engine oil-contaminated soil that was able to remove 44.55% and 30.43% of crude oil, respectively. *Pleurotus ostreatus* belonging to Basidiomycota carries genes required for the secretion of the ligninolytic enzymes implicated in the PAHs degradation^47^. However, a previous report corroborated that this remediator agent resulted in an incomplete PAH degradation in soil inoculated by this fungus. This drawback was attributed to a negative impact imposed by this fungus on the native soil bacteria required for the complete PAH degradation in soils^48^. However, we recovered a *Pleurotus* sp. in this study, but it does not show any promising PAH degradation ability in the assay repeated three times independently. We concluded that probably the materials (a-sexual stage) used to investigate its capacity in the culture medium are not an appropriate basis as this fungus showed promising results as a remediator agent once used as the sexual stage. More importantly, the gained fungus likely does not carry genes encoding metabolic pathways involved in the PAHs' degradation.

In this study, we identified *A. tenuissima* (5c-12) and *E. nigrum* (3b-1) as two potential isolates following a preliminary screening of 265 isolates obtained from crude oil non-contaminated and contaminated soils. Both isolates demonstrated high biodegradation efficiency when utilizing PAHs and petroleum as their sole carbon source (Figs. 2, 3). To the best of our knowledge, this is the first documentation of *A. tenuissima*'s ability to efficiently biodegrade petroleum-derived PAHs, both in vitro and in soil. A previous study by Abd et al.^49^ introduced *A. tenuissima* KM651985, isolated from marine decayed wood, as a novel source of laccase production. This strain exhibits tolerance to saline and alkaline conditions and has demonstrated efficient decolorization of two structurally diverse synthetic dyes, namely congo red and crystal viole^49^. It is worth noting that Laccases (benzenediol: oxygen oxidoreductases, EC 1.10.3.2) are blue multicopper oxidases catalyzing the oxidation of an array of aromatic substrates and are applied in various biotechnological processes such as the degradation of different recalcitrant compounds, bioremediation, dye decolorization, delignification of lignocellulosic, paper bleaching, and sewage treatment^50,51^. Furthermore, it was shown that tenuazonic acid (TeA), isolated from *A. tenuissima* MFP253011 extracts, had larvicidal activity against *Galleria mellonella*, the greater wax moth^52^. Previous research suggested that fungi indigenous to oil-contaminated soils may have developed multiple tolerance mechanisms for metabolizing oil compounds, potentially yielding less non-toxic substances compared to microorganisms from non-contaminated sites^25,53^. However, our findings contradict this notion, as we have validated that *A. tenuissima* (5c-12), recovered from a non-contaminated area (Table 2), exhibits remarkable biodegradation capabilities for PAHs. This fungal strain could serve as a promising agent for remediating soil exposed to oil spills. Our results align with the findings of Marchand et al.^19^, who demonstrated that long-term exposure to high PAH concentrations does not impair the biodegradation potential of microorganisms.

The results regarding the capacity of *Epicoccum nigrum* (3b-1) to utilize petroleum and PAHs as its exclusive carbon sources are in line with a previous report that highlights the fungus's efficient degradation of hydrocarbons found in different oils and gasoline. This fungus has been recognized as a promising candidate for mycoremediation in oil spill scenarios. Furthermore, it has been employed as a bioaugmentation agent in treating oily effluents within an air-lift reactor, resulting in a substantial 40% reduction in the initial oil and grease contents^54^. Indeed, both best-performing fungal individuals in the current study transform petroleum into simpler and harmless substrates, likely by employing extracellular enzymes and using them as energy sources. This ability enables them to have optimum growth in poor conditions and develop a mass of fungal growth in the bottom of the culture medium (Fig. 2), a phenomenon that has been indicated as a distinguishing feature of bioremediation’s ability of the crude-oil degrading fungal isolates^44^.

Studies have indicated that low molecular weight (LMW) PAHs, such as phenanthrene, are comparatively more volatile and water-soluble than high molecular weight (HMW) PAHs, rendering them more susceptible to biodegradation^8^. However, our results revealed that phenanthrene displayed a slight persistence based on the assessment of 12 fungal isolates’ ability to degrade four PAHs in vitro, as confirmed by precise GC/MS analysis (Fig. 3d). It is noteworthy that while most isolates demonstrated a substantial degradation of phenanthrene, the data depicted in Fig. 3d indicates a slightly lower degradation efficiency for phenanthrene (around 80–84%) compared to the other three tested PAHs, where the degradation ratio is within the range of 90–99%. Environmental concerns are typically heightened regarding HMW PAHs due to their long-term persistence in the environment, elevated toxicity, and mutagenic/carcinogenic properties. Among these, benzo(a)pyrene stands out as one of the most carcinogenic PAHs and is commonly used as a marker in exposure risk assessments. Interestingly, when evaluated under laboratory conditions, our top-performing remediators (5c-12 and 3b-1) exhibited the capability to biodegrade substantial quantities of HMW PAHs, including pyrene and benzo(a)pyrene, achieving a remarkable PAH degradation rate of 93–99% (Fig. 3).

In our soil assay, intentionally contaminating sterilized soil with petroleum, and using fungal inoculum prepared with wheat grains, both selected fungal isolates exhibited effective biodegradation of Total Petroleum Hydrocarbons (TPHs). Notably, inoculation with isolates 5c-12 and 3b-1 successfully degraded over 70% of long-chain hydrocarbons and the majority of petroleum fractions, including C8-C15 n-alkanes (Fig. 4, Supplementary Fig. 1). It's important to mention that we encountered challenges applying spore suspensions directly to the soil due to limited spore production by both isolates in the tested media. Previous research suggests that mycelial fragments are more efficient for PAH biodegradation than spore suspensions. Mycelial inoculum is crucial for establishing fungal presence in the soil, facilitating successful bioremediation. The elongation of hyphae allows mycelial fragments to effectively colonize the soil, enhancing the surface area for interactions with water-insoluble compounds and supporting PAH degradation. For instance, Potin et al.^55^ investigated 21 isolates obtained from soil in an old PAH-contaminated gasworks site and reported that *Coniothyrium* sp. (26.5%) and *Fusarium* sp. (27.5%) were highly effective in PAH degradation when applied as mycelial fragments in unsterilized soil.

In conclusion, our research demonstrates that *A. tenuissima* (5c-12) and *E. nigrum* (3b-1) exhibit the capability to efficiently degrade PAHs and petroleum compounds in both in vitro and soil settings. Notably, the effectiveness of these fungi in breaking down PAHs and petroleum appears to be consistent regardless of the initial contamination levels in the soil from which they were isolated. Utilizing these isolates as a fungal inoculum emerges as one of the most effective methods for bioremediating crude oil-contaminated soil. Furthermore, it is imperative to acknowledge that, due to practical constraints, our study concentrated solely on testing the two best-performing isolates in the soil assay. However, other isolates, particularly 1c-3 and 2a-22, have demonstrated significant potential in PAH biodegradation. While these isolates were not part of the current soil assay, we intend to evaluate their effectiveness in the soil environment in future studies. Moreover, bioremediation offers an efficient and cost-effective alternative to traditional physicochemical treatments. It is important to emphasize that mycoremediation, such as the application of these fungal isolates, is recommended for implementation in underdeveloped nations, as it can help mitigate risks, make land resources available for agricultural development, enhance food security, and contribute to environmental protection. Continued efforts should prioritize optimizing the formulation of the fungal inoculum to enhance the colonization and availability of these isolates in the soil, a crucial factor for the success of future experiments in this field.

### Supplementary Information

Supplementary Information.

## Data Availability

The raw data used and analysed during the present work are available from the corresponding author on request.
