# Supplementary material for: Potential biodegradation of polycyclic aromatic hydrocarbons (PAHs) and petroleum hydrocarbons by indigenous fungi recovered from crude oil-contaminated soil in Iran
Source: Sci Rep. 2023 Dec 13;13:22153. doi: 10.1038/s41598-023-49630-z (PMC10719355; doi:10.1038/s41598-023-49630-z)
Supplement: Supplementary file 1 — Supplementary Information. [file 41598_2023_49630_MOESM1_ESM.pdf]

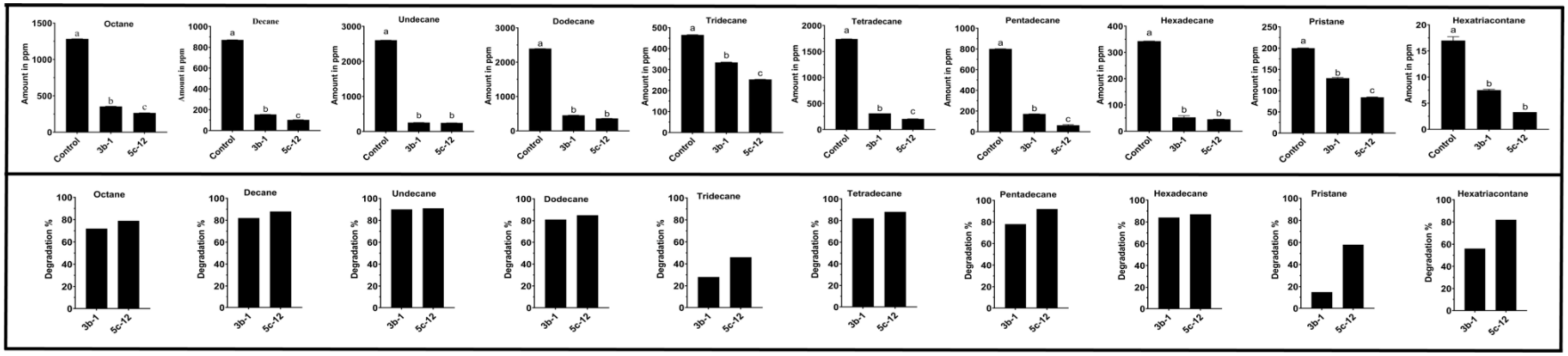

Supplementary Figure 1. Percentage of degradation of 10 compounds detected by GC-MS analysis in a soil assay after 60 days of incubation in petroleum-contaminated soil, inoculated with fungal strains 5c-12 (*Alternaria tenuissima*) and 3b-1 (*Epicoccum nigrum*), compared to a control (without fungi).

No		Fungal individual	Group
1	5c-12	*Alternaria tenuissima*	V
2	3b-1	*Epicoccum nigrum*	V
3	1c-3	*Neocamarosporium chichastianum*	IV
4	3a-46	*Penicillium chrysogenum*	III
5	3a-6-1	*Aspergillus terreus*	II
6	3a-3	*Botrytrichum piluliferum*	I
7	1a-7	*Alternaria chlamydospora*	II
8	1a-5-1	*Stachybotrys sp.*	IV
9	2a-33	*Epicoccum nigrum*	IV
10	1b-6	*Alternaria alternata*	IV
11	2a-22	*Penicillium oxalicum*	IV
12	3a-28	*Pleurotus floridanus*	I

Supplementary Table 1. Detailed information on the distribution of the 12 selected fungal isolates across different groups. Each row in the table represents a specific fungal individual, including its designation, taxonomic identification, and the assigned group.

Petroleum hydrocarbons (PHs) (ppm)	The residual contents of PHs in petroleum-contaminated soil, without any fungal cultures				The residual contents of PHs in petroleum-contaminated soil, inoculated with 3b-1 isolate				PHs degradation (%) by 3b-1	The residual contents of PHs in petroleum-contaminated soil, inoculated with 5c-12 isolate (3 replicates) and average				PHs degradation (%) by 5c-12
	**Rep 1**	**Rep 2**	**Rep 3**	**Average**	**Rep 1**	**Rep 2**	**Rep 3**	**Average**		**Rep 1**	**Rep 2**	**Rep 3**	**Average**	
**Octane**	**1297**	**1275**	**1280**	**1284**	**334**	**380**	**350**	**355**	**72**	**283**	**254**	**260**	**266**	**79**
**Decane**	**890**	**854**	**870**	**871**	**155**	**159**	**150**	**155**	**82**	**112**	**95**	**100**	**102**	**88**
**Undecane**	**2561**	**2657**	**2600**	**2606**	**236**	**278**	**250**	**255**	**90**	**238**	**257**	**240**	**245**	**91**
**Dodecane**	**2423**	**2375**	**2390**	**2396**	**406**	**503**	**450**	**453**	**81**	**391**	**331**	**360**	**361**	**85**
**Tridecane**	**452**	**484**	**460**	**465**	**314**	**359**	**330**	**334**	**28**	**259**	**251**	**250**	**253**	**46**
**Tetradecane**	**1778**	**1708**	**1740**	**1742**	**307**	**315**	**310**	**311**	**82**	**222**	**189**	**200**	**204**	**88**
**pentadecane**	**822**	**781**	**800**	**801**	**165**	**182**	**170**	**172**	**78**	**113**	**12.3**	**62**	**62**	**92**
**Hexadecane**	**350**	**339**	**340**	**343**	**97**	**8.20**	**53**	**53**	**84**	**47**	**43.2**	**46**	**45**	**87**
**Pristane**	**206**	**194**	**200**	**200**	**111**	**130**	**128**	**129**	**15**	**92**	**77**	**84**	**84**	**58**
**Hexatriacontane**	**14**	**20**	**17**	**17**	**8.**	**7**	**7.50**	**7.50**	**56**	**3.3**	**3.3**	**3.3**	**3**	**82**

Supplementary Table 2. Residual petroleum hydrocarbon (PH) content After 60 days in petroleum-contaminated soil inoculated with Isolates 3b-1 (*Epicoccum nigrum*), 5c-12 (*Alternaria tenuissima*), and control (without Fungi). Percentage of PH degradation by the two Isolates is reported.
